# Correlation of telomere length in brain tissue with peripheral tissues in living human subjects

**DOI:** 10.3389/fnmol.2024.1303974

**Published:** 2024-03-07

**Authors:** Annemarie J. Carver, Benjamin Hing, Benjamin A. Elser, Stephanie J. Lussier, Takehiko Yamanashi, Matthew A. Howard, Hiroto Kawasaki, Gen Shinozaki, Hanna E. Stevens

**Affiliations:** ^1^Department of Psychiatry, Carver College of Medicine, University of Iowa, Iowa City, IA, United States; ^2^Iowa Neuroscience Institute, Carver College of Medicine, University of Iowa, Iowa City, IA, United States; ^3^Interdisciplinary Graduate Program in Genetics, University of Iowa, Iowa City, IA, United States; ^4^Interdisciplinary Graduate Program in Human Toxicology, University of Iowa, Iowa City, IA, United States; ^5^Biostatistics Graduate Program, College of Public Health, University of Iowa, Iowa City, IA, United States; ^6^Department of Psychiatry and Behavioral Sciences, Stanford University School of Medicine, Standford University, Stanford, CA, United States; ^7^Division of Neuropsychiatry, Tottori University, Tottori, Japan; ^8^Department of Neurosurgery, Carver College of Medicine, University of Iowa, Iowa City, IA, United States; ^9^Hawk-Intellectual and Developmental Disabilities Research Center, University of Iowa, Iowa City, IA, United States

**Keywords:** brain, blood, buccal tissue, saliva, telomere, neuroscience, neuropsychiatric disorders, neurology

## Abstract

Telomeres are important to chromosomal stability, and changes in their length correlate with disease, potentially relevant to brain disorders. Assessing telomere length in human brain is invasive, but whether peripheral tissue telomere length correlates with that in brain is not known. Saliva, buccal, blood, and brain samples were collected at time points before, during, and after subjects undergoing neurosurgery (*n* = 35) for intractable epilepsy. DNA was isolated from samples and average telomere length assessed by qPCR. Correlations of telomere length between tissue samples were calculated across subjects. When data were stratified by sex, saliva telomere length correlated with brain telomere length in males only. Buccal telomere length correlated with brain telomere length when males and females were combined. These findings indicate that in living subjects, telomere length in peripheral tissues variably correlates with that in brain and may be dependent on sex. Peripheral tissue telomere length may provide insight into brain telomere length, relevant to assessment of brain disorder pathophysiology.

## 1 Introduction

Telomeres are hexanucleotides tandem repeats present at the end of chromosomes that maintain chromosome stability (Bailey and Murnane, 2006). These repeats can vary in length in the DNA between different tissues (Demanelis et al., 2020) and shorten over time with cell replication (Victorelli and Passos, 2017) which is associated with cellular senescence and apoptosis (Victorelli and Passos, 2017). Additionally, telomere length is maintained by the shelterin complex (Xin et al., 2008). Significant research has shown that telomere length (TL) can be a reliable biomarker for a variety of age-related diseases including osteoporosis and infertility (Vasilopoulos et al., 2019; Kakridonis et al., 2023; Tsatsakis et al., 2023). Although TL in cells from saliva and blood shorten with biological factors such as age (Saretzki, 2018), TL can also be affected by environmental factors such as psychosocial stressors (Rentscher et al., 2020) and in stress-related diseases, including neuropsychiatric disorders (Vakonaki et al., 2018) in which inflammation and oxidative stress may reduce the integrity of the shelterin complex (Zhang et al., 2007). For example, previous systematic reviews observed significant shortening of blood leukocyte TL in dementia, multiple sclerosis, and across neuropsychiatric disorders including mood and anxiety disorders, PTSD, schizophrenia, and other psychotic disorders (Darrow et al., 2016; Buhring et al., 2021; Fu et al., 2022). Brain TL is altered in individuals diagnosed with neuropsychiatric disorders in postmortem samples (Mamdani et al., 2015) and may be involved mechanistically in these brain disorders. Brain TL affects neuronal processes inherent to cognitive, motor, and socioemotional functioning such as neuronal differentiation and maturation (Ferron et al., 2009). Similarly, associations between shortened leukocyte TL correlates with and may reflect mechanisms of risk for other diseases and mortality including in cardiovascular, respiratory, digestive, musculoskeletal, and infectious domains (Schneider et al., 2022). Although shortened TL has more frequently been associated with increased disease risk, some studies have observed increased TL with childhood Attention Deficit/Hyperactivity Disorder symptoms (Momany et al., 2021) and cancer (Lin and Epel, 2022). Together, these studies highlight the potential role of TL in health, both as a biomarker and as a potential pathophysiological target for intervention.

**Figure 1:**
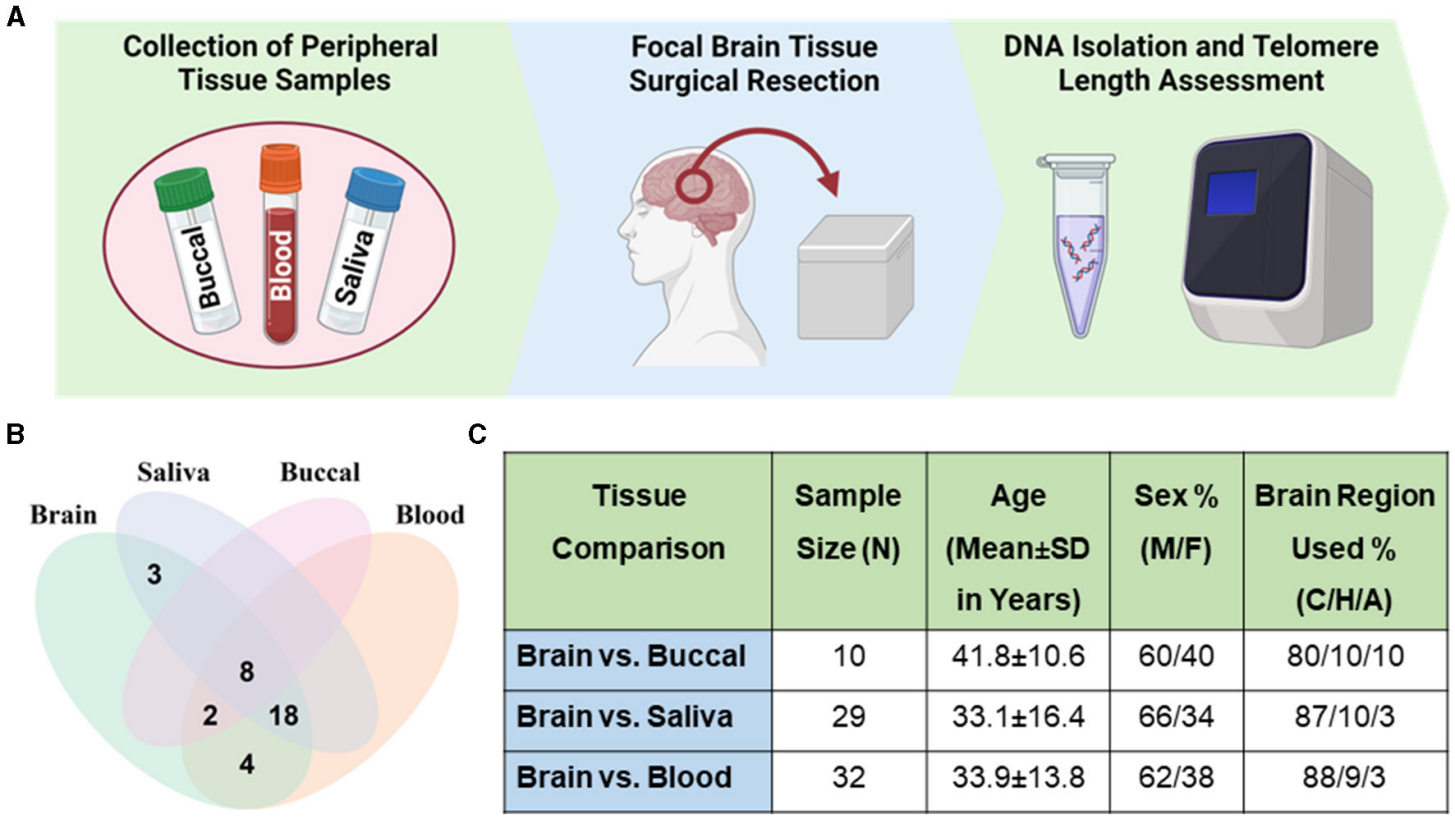
Sample demographics. **(A)** Workflow schematic of sample collection and processing. Panel created with BioRender.com. **(B)** Venn diagram of samples of brain and peripheral tissues across patients. Thirty-five patients had brain and at least one peripheral tissue sample collected. **(C)** Table describing samples with brain-peripheral tissue pairs. Percentage of male (M) and female (F) samples. Percentage of brain region used from sample group listed as cortex (C), hippocampus (H), and amygdala (A).

While peripheral tissue TL may provide an indication for neuropsychiatric disease, understanding potential telomere pathophysiological mechanisms in the brain directly relevant to neuropsychiatric diseases would require invasive sampling. Brain TL biology and that of peripheral tissues more easily sampled as indicators of disease may be similar, despite distinct differences in the cells' replicative potential and other processes. Little is known about how TL from peripheral cells might reflect those in the brain. One recent study assessed TL across more than 20 different postmortem tissues in 952 individuals (Demanelis et al., 2020). This study showed that TL varies across different tissues and that whole blood TL moderately correlates with TL in brain cortical samples (Demanelis et al., 2020). In addition, other studies have observed correlation between leukocytes TL and brain imaging phenotypes of neurodegenerative diseases (Cao et al., 2023; Topiwala et al., 2023). As such, TL from peripheral tissues have been shown to reflect changes in the brain. A limitation of these studies is that commonly used peripheral tissues as possible indicators of disease, such as saliva and buccal samples, were not included. As such, the relationship of average TL in these commonly sampled peripheral tissues with brain TL was evaluated in the present study using standard qPCR methods for average telomeric repeats relative to a single gene copy (T/S ratio). This study aims to evaluate the relationship between commonly sampled peripheral tissues and brain TL from 35 living patients with medically intractable epilepsy who underwent focal brain tissue surgical resection (Figures 1A– C).

## 2 Materials and methods

### 2.1 Patient recruitment

Subjects with medically intractable epilepsy undergoing neurosurgery were recruited for this study at the University of Iowa Hospitals and Clinics. This study was approved by the University of Iowa's Human Subjects Research Institution Review Board. Study participants were enrolled as described in our previous studies (Braun et al., 2019; Yamanashi et al., 2021). Briefly, written informed consent was obtained. Pathology reports of resected brain tissues showed samples with a range of pathologies, including gliosis, sclerosis, and focal cortical dysplasia, but samples with neoplasm were excluded due to potential impacts on TL. Pre-surgical blood, saliva, and buccal samples were collected in the operating room prior to collection of brain samples during surgery. In a few instances, samples collected after surgery were used in the analysis when pre-surgical samples were not available or were excluded for technical problems; post-surgical blood samples were collected in the operating room and buccal and saliva were collected by patients at home and set in by mail. Thirty-five patients had a brain sample and at least one peripheral tissue sample collected. All three peripheral tissues were not able to be collected from every patient. This is especially reflected in the lower number of buccal tissue samples, as collection of this tissue was added later during the study.

### 2.2 Tissue collection, DNA extraction, and telomere length measurement

Saliva was collected from participants following the protocol from Oragene OG500 collection kit (DNA Genotek Inc., OGR-500, Ottawa, Ontario, Canada). Buccal was collected using buccal swabs (Puritan 25–1506 1PF TT MC). Cells were concentrated in microcentrifuge tubes. Venous whole blood samples were collected in EDTA tubes. Resected brain tissue samples were immediately stored and transported on dry ice and a portion of each brain region was sent to pathology. All samples were stored at −80°C until DNA extraction. DNA was extracted using MasterPure Complete DNA and RNA purification kit as per manufacturer's instructions and quantified using Qubit fluorometry (ThermoFisher Scientific, Waltham, MA, USA).

### 2.3 Telomere length assessment

Telomere length was assessed as previously described (Momany et al., 2021). Samples were deidentified so that they could be run blindly by experimenters. Briefly, qPCR was performed using 1X Power SYBR green together with 33 ng of DNA, 900 nM of telomere (T) primers (Telc: TGTTAGGTATCCCTATCCCTATCCCTATCCCTATCCCTAACA, Telg: ACACTAAGGTTTGGGTTTGGGTTTGGGTTTGGGTTA GTGT) and 600 nM of single (S) copy albumin gene primers (Albu: CGGCGGCGGGCGGCGCGGGCTGGGCGGAAATGCTGCACA GAATCCTTG, Albd: GCCCGGCCCGCCGCGCCCGTCCCGCC GGAAAAGCATGGTCGCCTGTT) per reaction. Samples were run in triplicates and sample cycle threshold values (C_T_) were averaged for each sample. All experimental plates were also performed in duplicates with sample position reversed. Individual samples were required to have an intraplate coefficient of variation of <0.1 and inter-plate coefficient of variation of <0.6. All samples passed this quality control criteria. T/S Ratio was calculated using the ΔC_T_ method (2^−|*Ct*(*T*)−*Ct*(*S*)|^) as previously described (Momany et al., 2021). Quantitative PCR was performed on the Viia7 (Thermofisher) with thermal cycling profile adapted from previously published protocol (Schneider et al., 2022). Stage 1: one cycle of 50°C for 2 min to activate Taq polymerase followed by 95°C for 10 min. Stage 2: two cycles of 94°C for 15 s followed by 49°C for 15 s. Stage 3: 32 cycles of 94°C for 15 s followed by 62°C for 10 s and 74°C for 15 s. Melt curve detection: 1 cycle 95°C for 15 s, 60°C for 1 min followed by 95°C for 15 s. Analysis of melt curve showed a single product detected from the amplification.

### 2.4 Analysis

All statistical analysis was performed using PRISM or R. To assess any TL differences in different brain region samples, a ROUT outlier test followed by a one-way ANOVA was performed. When available, cortical brain samples were used in cross-tissue correlations. Similarly, to determine any differences between pre- and post-surgical peripheral tissue sample TL, a ROUT outlier test followed by Wilcoxon matched-pairs signed rank test was performed on these data. When available, peripheral tissues collected pre-surgery were used in cross-tissue correlations. For correlations across subjects of brain TL and each peripheral tissue sample TL, outliers were identified using a ROUT outliers test. One outlier was removed from Buccal vs. Brain analysis. Four outliers were removed from each of the Blood vs. Brain and Saliva vs. Brain analyses. After exclusion of outliers, partial correlation was performed controlling for age to compute Pearson correlation coefficient; age was used as a covariate since peripheral TL shortens across the lifespan (Demanelis et al., 2020). Correction for multiple testing was performed by false-discovery rate (FDR).

## 3 Results

### 3.1 Tissue sample telomere length

Cortical samples were the most commonly collected brain region (86%), so they were used for analysis in most cases, followed by samples of hippocampus (10%) or amygdala (4%), when cortex was unavailable. No significant difference was found in the average TL in samples from these three brain regions (one-way ANOVA; *P* = 0.3901; Supplementary Figure 1A). Average TL of samples collected pre- or post-surgery were not significantly different (buccal; *FDR* = 0.6875, saliva; *FDR* = 0.1641, blood; *FDR* = 0.6875; Supplementary Figures 1B–D). The fold change between pre- and post-surgery samples were 1.1 ± 0.8, 0.9 ± 1 and 1.8 ± 1.7 (mean ± SD) for blood, saliva and buccal respectively. For subsequent cross-tissue correlation analysis, we included four subjects with post-surgical blood and three subjects with post-surgical buccal samples.

### 3.2 Comparison of peripheral tissue to brain telomere length

**Figure 2:**
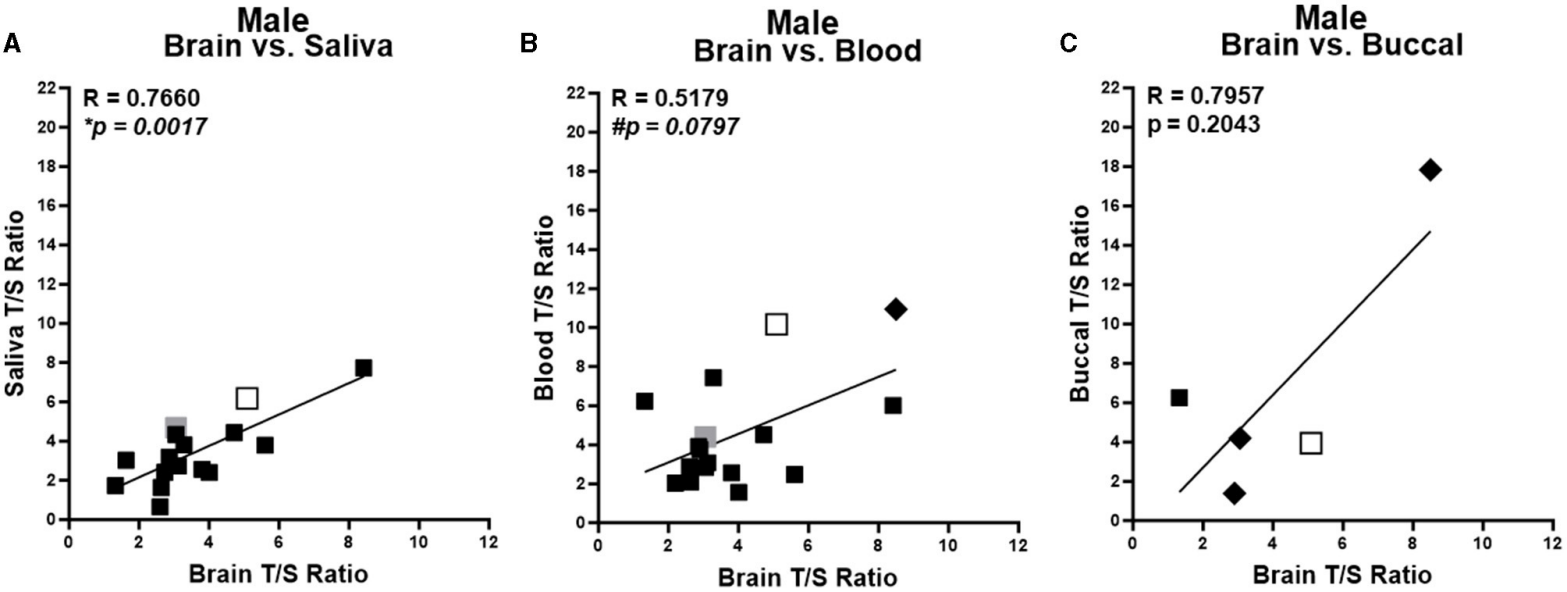
Correlation of telomere length between brain and peripheral tissues in males. Correlation of average telomere length (T/S ratio) across individuals between brain and peripheral tissues for males. Brain region in each sample specified by marker color: black = cortex; open shape with black border = hippocampus; and gray = amygdala. Pre-surgery collected peripheral samples are squares and post-surgery collected peripheral tissues are diamonds. **(A)** Brain compared to saliva samples. **(B)** Brain compared to blood samples. **(C)** Brain compared to buccal samples. *Corrected *P* < 0.05, ^#^corrected *P* < 0.1. *R* values were generated from partial correlation controlling for age; *P*-values are corrected for multiple testing by FDR.

Due to sex differences in TL (Lansdorp, 2022; Mendez-Chacon, 2022), we first performed cross-tissue correlations stratified by sex. With more samples collected from male subjects (*n* = 22; 63%), a more robust analysis was possible in males. Males showed a significant positive correlation of average TL from brain compared with saliva samples (Figure 2A) and a trending significant correlation of brain compared with blood samples (Figure 2B). There was no significant correlation of average TL of brain samples and the small number of buccal samples (Figure 2C). Within female samples, no significant correlations of average TL were found for brain and any of the peripheral tissue samples (Supplementary Figures 2A–C).

We also evaluated male and female samples together in an overall analysis, no significant or trending correlation was identified between saliva or blood and brain average TL (Supplementary Figures 3A, B). When both male and female samples were evaluated together, a significant positive correlation was found between brain and buccal sample average TL (Supplementary Figure 3C).

## 4 Discussion

Overall, the current study suggests that average TL in different peripheral tissues varies in correlation with average TL in the brain across individuals. Within the larger number of male subjects, brain TL correlated with saliva TL, demonstrating a significant positive correlation. Additionally, there was a trending positive correlation between brain and blood TL. This indicates that saliva and possibly blood could be useful indicators within males for brain TL. No significant correlations were identified in the smaller number of female subjects. When both male and female samples were analyzed together, the smallest set of samples of buccal TL positively correlated with brain TL, while saliva and blood TL did not. However, given the small number of samples of buccal tissue and in the study overall, results should be interpreted with caution.

Differences in buccal and saliva TL correlation with brain may result from different proportions of cell types in each sample source. Epithelial cells make up a greater proportion of cells in buccal samples vs. leukocytes in saliva (Theda et al., 2018) which may result in a different relationship to brain TL. As buccal is also an easily collected and non-invasive tissue, more research should be performed to identify the correlation of this tissue to brain TL especially due to the promising significant positive correlation seen when both sexes were used for analysis.

Although the current study suggests that in males, saliva average TL may reflect average TL in the brain, there are several limitations to be noted. First, our sample sizes are small which impacts the power and representativeness of our assessments, particularly in the analyses stratified by sex. However, in the analyses with the most samples (male comparisons of brain with saliva and blood), correlations for these pairings of tissues were significant or trending significant and similar, convergence which adds support to the hypothesis that condition of the brain may be reflected by peripheral TL. For brain comparisons with saliva and blood, females (*n* = ~10) had approximately half the sample size of males (*n* = ~ 20) which reduced the power of the female analysis. It is known that females on average have longer telomere lengths than males likely due to differences in estrogen and dyskerin that may contribute to differences in lifespan (Lansdorp, 2022; Mendez-Chacon, 2022), and this may contribute to some of the variability seen in our study. It is also possible that female samples had more inter- or intra-individual unmeasured heterogeneity, from the influences of cycling hormones or other sources.

Few opportunities exist to obtain brain tissue from living subjects, so all samples in this study were collected from individuals with medically intractable epilepsy which is another limitation to generalizability. There are mixed results previously on the association of peripheral TL with epilepsy: one study found increased telomere shortening in patients with drug-resistant epilepsy (Miranda et al., 2020) and another study identified no TL differences in individuals with epilepsy (Luo et al., 2023). Another study shows that individuals with the rare disorder, Revesz syndrome, that often experience seizures display decreased ability of the shelterin complex to protect telomeres from shortening (Karremann et al., 2020). These studies indicate that some individuals with epilepsy may have impaired protection of telomere length by the shelterin complex, although preclinical studies also suggest the seizures may induce telomerase expression which may increase TL (Fu et al., 2022). Peripheral TL has been found to be related to epilepsy as with other disorders (Miranda et al., 2020), but our results may not reflect TL correlations in individuals without epilepsy.

In summary, in living human subjects, buccal TL provided the highest correlation with brain TL relative to saliva and blood TL. This study is unique as it allows for the rare insight into telomere length from live human brain tissue to be compared to peripheral tissues from the same individuals. Due to multiple limitations of the size and nature of the subjects in this study, this finding may not generalize to other human cohorts. To expand upon this study, further research should be done to identify if sex differences may be present in the correlation of peripheral tissue TL with brain TL. These findings expand on previous studies, as three commonly collected peripheral tissues were included, two of which were not included in a previous large postmortem data set comparing TL in brain and other tissues. Future studies should be performed with a larger sample size to validate this finding.

## Data availability statement

The original contributions presented in the study are publicly available. This data can be found at: Iowa Research Online: https://iro.uiowa.edu/esploro/outputs/dataset/Human-brain-and-peripheral-telomere-length/9984465954302771/filesAndLinks?forceView=true&mode=quickaccess&index=0.

## Ethics statement

The studies involving humans were approved by University of Iowa's Human Subjects Research Institution Review Board. The studies were conducted in accordance with the local legislation and institutional requirements. Written informed consent for participation in this study was provided by the participants' legal guardians/next of kin.

## Author contributions

AC: Data curation, Formal analysis, Funding acquisition, Investigation, Methodology, Visualization, Writing – original draft, Writing – review & editing. BH: Conceptualization, Data curation, Formal analysis, Investigation, Methodology, Project administration, Visualization, Writing – original draft, Writing – review & editing. BE: Data curation, Formal analysis, Funding acquisition, Investigation, Methodology, Writing – review & editing. SL: Data curation, Formal analysis, Investigation, Methodology, Writing – review & editing. TY: Conceptualization, Data curation, Writing – review & editing, Resources, Investigation. MH: Conceptualization, Resources, Writing – review & editing, Investigation. HK: Conceptualization, Resources, Writing – review & editing, Investigation. GS: Conceptualization, Data curation, Funding acquisition, Investigation, Project administration, Supervision, Writing – review & editing, Resources. HS: Funding acquisition, Investigation, Project administration, Supervision, Writing – original draft, Writing – review & editing, Conceptualization, Data curation, Resources.
