# Supplementary material for: Correlation of telomere length in brain tissue with peripheral tissues in living human subjects
Source: Front Mol Neurosci. 2024 Mar 7;17:1303974. doi: 10.3389/fnmol.2024.1303974 (PMC10954899; doi:10.3389/fnmol.2024.1303974)
Supplement: Supplementary file 1 [file Data_Sheet_1.pdf]

Supplementary Material

1 Supplementary Figures

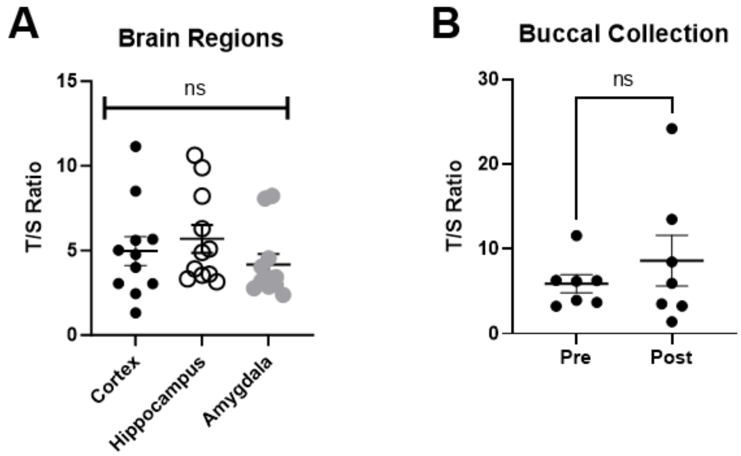

Supplemental Figure 1: Controlling for sample collection variation. (A) Brain telomere length (T/S ratio) collected from three regions (cortex, hippocampus, and amygdala) compared with one-way ANOVA. No significant difference was found between the three regions. **(B-D)** Peripheral tissues T/S ratio from collections prior to (Pre) or post (Post) surgery were compared. No difference is seen in Pre or Post collected tissues in **(B)** buccal, **(C)** saliva, and **(D)** blood. ns; not significant.

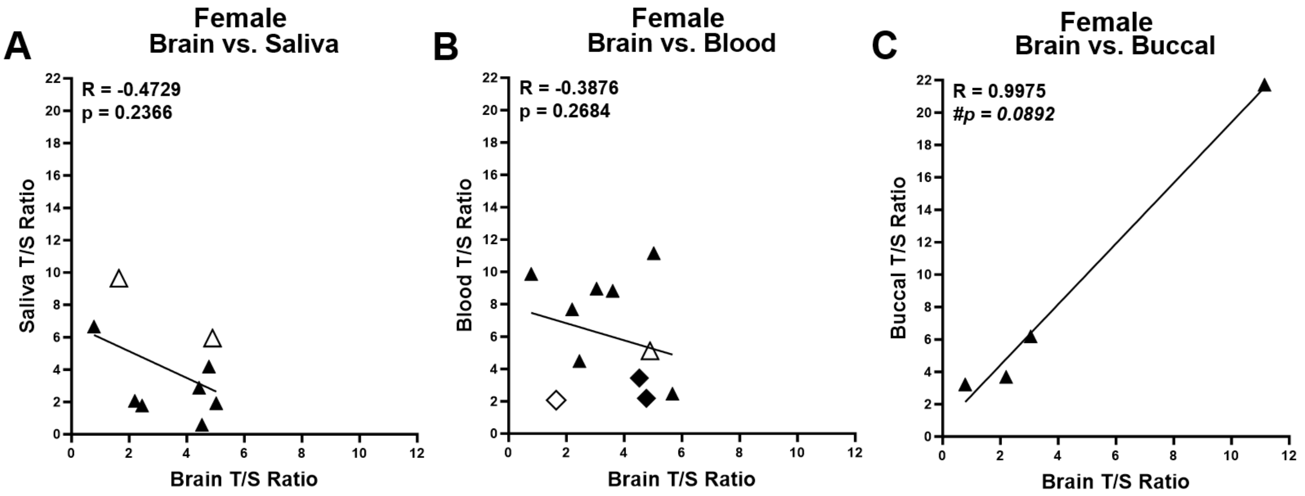

Supplemental Figure 2: Correlation of telomere length between brain and peripheral tissues in females. Correlation of average telomere length (T/S ratio) across individuals between brain and peripheral tissues for female samples. Brain region in each sample specified by marker color: Black = cortex; open shape with black border = hippocampus. Pre-surgery collected peripheral samples are triangles and post-surgery collected peripheral tissues are diamonds. **(A)** Brain compared to saliva samples. **(B)** Brain compared to blood samples. **(C)** Brain compared to buccal samples. * = corrected P < 0.05, # = corrected P < 0.1. R values were generated from partial correlation controlling for age; p-values are corrected for multiple testing by FDR.

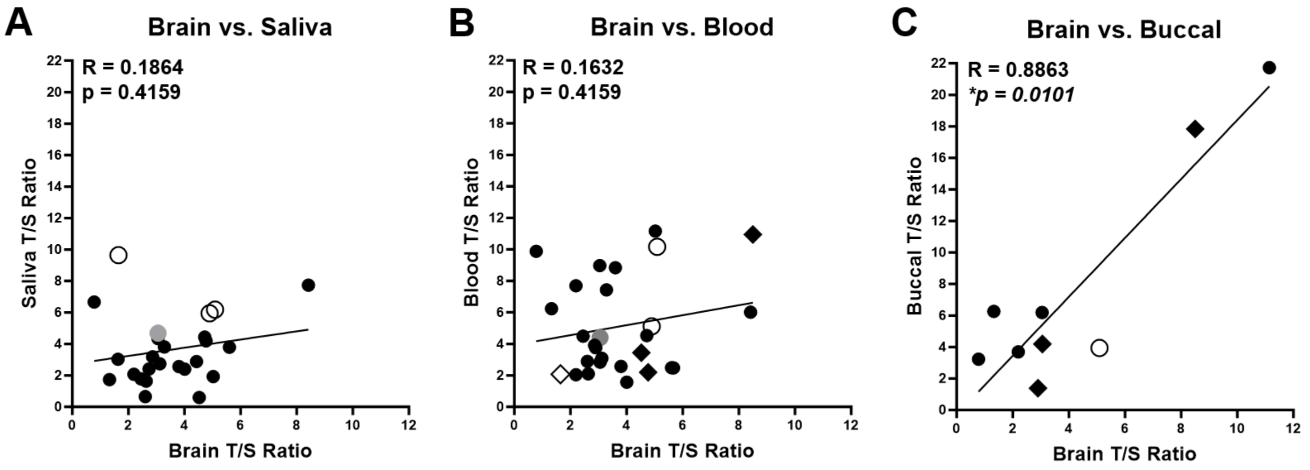

Supplemental Figure 3: Correlation of telomere length between brain and peripheral tissues regardless of sex. Correlation of average telomere length (T/S ratio) across individuals for **(A)** brain compared to buccal samples, **(B)** brain compared to saliva samples **(C)** brain compared to blood samples. Brain region in each sample specified by marker color: black = cortex; open shape with black border = hippocampus; and grey = amygdala. Pre-surgery collected peripheral samples are circles and post-surgery collected peripheral tissues are diamonds. * = corrected P < 0.05. R values were generated from partial correlation controlling for age; p-values are corrected for multiple testing by FDR.
